# Case of Diseased Uterus

**Published:** 1840-03-07

**Authors:** Geo. Hamilton


### CASE OF DISEASED UTERUS.

BY GEO. HAMILTON, M. D.

To the Editors of the Medical Examiner.

*Gentlemen*:—If the following case of diseased uterus be thought worthy of publication, it is at your disposal. GEO. HAMILTON.

*Centreville, New Castle Co., Del., Feb.* 25, 1840.

On the 29th of January, 1839, I was requested to visit Margaret (aged 27) wife of Timothy Doud, then residing in Kennet Township, Chester Co., Pa. On arriving there, I found her suffering from labour pains attended with a moderate sanguineous discharge from the vagina, threatening abortion, which, in fact, took place on the 31st, and differed in no respect in the course of symptoms attending it, from the majority of such cases, excepting the greater amount of pain experienced before the embryo was expelled. She recovered rapidly, and was soon able to attend to her household affairs. This was her first pregnancy. On the 24th April, (about eleven weeks from her first abortion) I was again sent for, and again found her labouring under severe contractile pain of the uterus, brought on, as she thought, by too great exertion whilst engaged in whitewashing. On this occasion, there was no discharge from the vagina, but the hardening of the uterus during each contraction, could be readily felt by placing the hand over that organ. I considered her about two months advanced in gestation, and this corresponded with her own opinion of her situation. With a view to quiet the efforts of the uterus she was bled, and an opiate given, which soon afforded so much relief, that after directing the precautionary measures requisite in such cases, I left her.

In two days after, the pain returned, and was removed by the means before employed. The disposition to abort, however, still continued, inasmuch as there was a return of contractile pain every few days. After the fourth attack of severe pain, the contractions of the uterus became less powerful, and more irregular in their recurrence, sometimes happening every day, or even several times during the day. About the same time, a sub-acute inflammation of the uterus occurred, apparently induced by the violent and repeated contractions of that viscus, thus adding greatly to the difficulty of the case. Means were now resorted to for subduing the inflammation, and consisted principally of bleeding, cupping, and blistering; the use of gentle laxatives, sedatives, and strict attention to diet, and quietude in the horizontal posture. These measures had the desired effect so long as the uterus remained quiescent, but no sooner was it thrown into action, than whatever advantage might have been gained over the inflammation, was then lost, and the patient reduced to a still more unfavourable situation.

She continued in this way for two or three weeks, during which time the tenderness over the uterus, and symptomatic fever held a constant relation to the amount of contractile pain experienced. Her situation I now thought extremely critical, and every day appeared to render it still more so. What was to be done? It occurred to me that abortion held out by far the most likely method of salvation to the woman, provided that the vital powers of her system could withstand that process. The immense pain she suffered during the miscarriage that took place in January, and before alluded to, satisfied me that, in her present situation, this was impracticable, and that being the case, she seemed destined, sooner or later, to sink from the effect of local disease, and irritative fever. At this juncture of affairs, a consultation was decided upon, and Dr. Thomas Seal, of Unionville, Chester County, was requested to see the patient. He advised a steady perseverance in the use of the means before employed, as the most likely to produce relief; consequently they were continued for a while longer, with about the same result as heretofore. Our patient was now (June 10th) much reduced in flesh and strength, her pulse became more irritated, and no decided advantage having been obtained from the course of treatment hitherto pursued, we concluded to venture upon the use of spt. turpentine internally, and applied by means of flannel externally, with a more free administration of laxative medicines, than we had before ventured to give, lest we should increase the abortive pain. By these means the symptoms gradually improved, so that on the 23d of June, the day I last saw her, she had walked about the room, said she was free from pain, or nearly so, and expressed a strong desire to be removed to her mother's house in about a week from that time, to which I assented, provided she continued to improve. As I have since been informed, she took worse the day after I saw her, but when the time fixed upon for removing came, she went, and appeared to bear the journey (about 18 miles) well; as she was able to take supper with the family after her arrival there, and appeared rather lively during that evening. On the next day, she was taken with her old symptoms, and so continued better and worse for five or six days, when suddenly she began to sink rapidly, and in about three hours expired. Dr. Dilworth, of Oxford, (to whose vicinity she was removed) informs me by letter, that he was called upon to see her a short time before her decease, and supposed her to be flooding, but upon inquiry, ascertained that nothing of this kind had occurred. I am indebted to Dr. Dilworth for an account of the post mortem examination he made, which, in substance, is as follows. On making the necessary incision, and exposing the abdominal cavity, a mass of coagulated blood presented itself, which, upon examination, was found to be equally diffused over nearly the entire surface of the bowels, reaching from the lower part of the abdomen, nearly to the margin of the liver. Upon removing this, and cautiously approaching the uterus, this organ was found enlarged to about the size of the fourth month of gestation.

The fundus uteri, for about three inches in diameter, was in a decomposed or mortified state, and partially separated from the less diseased portion, from an enlarged vessel or vessels from which the hæmorrhage appeared to have proceeded. The disorganized portion being removed, the uterine cavity was found to be filled with a semi-transparent substance, composed of small globules, bearing some resemblance to the vitreous humour of the eye. Except the parts already mentioned, the contents of the abdomen were in a sound condition.

